# Induction of Premature Labor by Uterine Catheterism

**Published:** 1860-02

**Authors:** 

**Affiliations:** Vienna


### INDUCTION OF PREMATURE LABOR BY UTERINE CATHETERISM.

BY PROF. BRAUN, OF VIENNA.

(From the Nor. Amer. Chir. Reviews.)

Professor Braun, of Vienna, states that he has for several years given a preference to this mode of inducing premature labor; inasmuch as it is very certain, operates rapidly and safely, brings on the pains with gradual energy, gives rise to no ill consequence, such as congestion or injury of the uterus, or detachment of the placenta, and is performed by the simple application of a simple instrument. One disadvantage of the procedure is, that the membranes may become somewhat easily ruptured, especially in primiparæ. In order to prevent this accidental rupture, the author softens in hot water the end of a well-oiled catgut bougie, a foot long, and from two to three lines thick, and passes it along the index finger with a twisting movement to the uterine cavity, until only a portion, equal to two fingers' breadths, remains in the vagina. The bougie so passed always excites pains in from six to twenty hours, does no injury to the membranes, and is to be removed only just before the discharge of the waters, or the birth of the child. The employment of a gum catheter, having a very thin, flexible stilette, is usually also attended with good effect. Its application is difficult, however, when the vagina is narrow, and deviates from the pelvic axis. During the session 1857–8, Professor Braun employed catheterism twelve times, nine children being born alive, and six dead—three being twin-births. Of the mothers, eight recovered, and four died during the puerperal state, pneumonia, tubercle, and Bright's disease having been respectively the causes of death. The labor was terminated at an average period of twelve hours after the introduction of the catheter. Brief accounts of these twelve cases were subjoined.

### IODIDE OF POTASSIUM AS AN ANTIGALACTIC.

BY PROFESSOR ROUSSET.

We extract the following from the *North American Chirurgical Review*, translated from the *Journal de Bordeaux*.

"The troublesome milk-knots which tend to appear especially at the commencement of lactation, giving rise to fever, inflammation of the breast, and abcesses, indicate a diminution of the secretion of milk by therapeutic means. As the usual measures (emollient cataplasms, dieting, and laxatives) had frequently proved insufficient, the author tried the iodide of potassium. The results were as follows:—The iodide of potassium occasions a considerable decrease of the milk, and in consequence prevents and remove milk-knots, particularly if at the same time the child is put to the breast. The milk returns quickly, if the medicine is not used any longer than two to three days; its effect is more decided if the dose does not exceed forty to fifty centigrammes daily. The secretion of milk can be prevented almost completly if the iodide of potassium is given on the first or second day after delivery. The author gives a full report of seven cases to confirm the above statements.

